# Heterozygous p53-R280T Mutation Enhances the Oncogenicity of NPC Cells Through Activating PI3K-Akt Signaling Pathway

**DOI:** 10.3389/fonc.2020.00104

**Published:** 2020-02-05

**Authors:** Zhen-Qi Qin, Qi-Guang Li, Hong Yi, Shan-Shan Lu, Wei Huang, Zhuo-Xian Rong, Yao-Yun Tang, Zhi-Qiang Xiao

**Affiliations:** ^1^Department of Otolaryngology Head and Neck Surgery, Xiangya Hospital, Central South University, Changsha, China; ^2^Research Center of Carcinogenesis and Targeted Therapy, Xiangya Hospital, Central South University, Changsha, China; ^3^The Higher Educational Key Laboratory for Cancer Proteomics and Translational Medicine of Hunan Province, Xiangya Hospital, Central South University, Changsha, China

**Keywords:** nasopharyngeal carcinoma, p53, R280T mutation, oncogenicity, Akt

## Abstract

A heterozygous point mutation of p53 gene at codon 280 from AGA to ACA (R280T) frequently occurs in nasopharyngeal carcinoma (NPC) cell lines, and about 10% NPC tissues. However, the role of this mutation in the pathogenesis of NPC remains unclear. In this study, we generated p53 knockout (KO) NPC cell lines from CNE2 cells carrying heterozygous p53 R280T (p53-R280T) mutation and C666-1 cells carrying wild-type p53 by CRISPR-Cas9 gene editing system, and found that KO of heterozygous p53-R280T significantly decreased NPC cell proliferation and increased NPC cell apoptosis, whereas KO of wild-type p53 had opposite effects on NPC cell proliferation and apoptosis. Moreover, KO of heterozygous p53-R280T inhibited the anchorage-independent growth and *in vivo* tumorigenicity of NPC cells. mRNA sequencing of heterozygous p53-R280T KO and control CNE2 cells revealed that heterozygous p53-R280T mutation activated PI3K-Akt signaling pathway. Moreover, blocking of PI3K-Akt signaling pathway abolished heterozygous p53-R280T mutation-promoting NPC cell proliferation and survival. Our data indicate that p53 with heterozygous R280T mutation functions as an oncogene, and promotes the oncogenicity of NPC cells by activating PI3K-Akt signaling pathway.

## Introduction

Nasopharyngeal carcinoma (NPC) arises from the epithelial lining of the nasopharynx (1). It has a high prevalence in southern China, Southeast Asia, northern Africa and Alaska, with remarkable ethnic and geographic distribution (2). The annual incidence rate reaches 25 cases per 100,000 people in the endemic regions, which is about 25-fold higher than that in the rest of the world, posing one of the most serious public health problems in these areas (2). NPC is closely associated with the Epstein-Barr virus (EBV) infection and genetic susceptibility (3). Familial clustering of NPC has been observed not only in the southern Chinese population but also in the non-chinese, low-risk populations (4). It suggests that genetic alterations of tumor suppressor genes and proto-oncogenes may be important in NPC carcinogenesis.

P53 is a sequence-specific DNA binding protein, which consists of two N-terminal transactivation domains, a central DNA binding domain (DBD), a C-terminus including nuclear localization signals and an oligomerization domain needed for transcriptional activity (5). As the “guardian of the genome,” p53 is important to suppress cancer development and progression. P53 mutations are observed in approximately half of the human cancers, most of which occur in the region encoding p53's DBD and lead to disordered p53 signaling pathway (6–8). P53 mutations often result in accumulation of the mutant p53 protein, which either loses tumor suppressor function or gains oncogenic activity. R280 residue located in the DBD of p53 gene plays an important role in DNA recognition and p53-DNA complex stability (9). In the p53 mutation database established by IARC, p53 mutation at codon 280 (R280T) was found in tumors originating from 30 types of human tissues such as bladder, breast, nasopharynx, accessory sinus and mouth larynx, and in a few tumor cell lines such as NPC, bladder carcinoma, breast carcinoma, gastric, and esophageal cancer cell lines (10). For NPC, the prevalence of p53 mutations is about 30% (11–16). Among them, a heterozygous point mutation of p53 gene at codon 280 from AGA to ACA (Arg changed to Thr) (R280T) was identified in the five NPC cell lines (CNE1, CNE2, TW06, TW01, and HONE1), with a mutation rate of about 10% in NPC tissues (15, 17–19). However, the functions of this heterozygous p53-R280T mutation in NPC remain unclear.

In this study, we generated p53 knockout NPC cell lines from CNE2 carrying heterozygous p53-R280T mutation (17) and C666-1 carrying wild-type (wt) p53 (15) using the CRISPR-Cas9 gene editing system. We found that knockout of heterozygous p53-R280T inhibited while knockout of wt p53 increased the oncogenicity of NPC cells. To explore the mechanism of heterozygous p53-R280T-promoting NPC cell oncogenicity, we compared the mRNA expression profiles in the heterozygous p53-R280T knockout and control CNE2 cells by mRNA sequencing, and found PI3K-Akt signaling pathway involved in the tumor-promotion effect of heterozygous p53-R280T mutation.

## Materials and Methods

### Knockout of p53 in NPC Cell Lines Using the CRISPR-Cas9 Gene Editing System

Human NPC cell lines (CNE2, C666-1) were cultured and maintained in RPMI-1640 medium containing 10% (v/v) fetal bovine serum (FBS) (Thermo, USA) at 37°C. For p53 knockout, the guide RNA (gRNA) sequence was GCAGTCACAGCACATGACGG, which was designed using the website software from Massachusetts Institute of Technology (USA) (https://zlab.bio/guide-design-resources/). CNE2 and C666-1 cells were transfected with the plasmid pGK1.1 containing p53 gRNA and control vector pGK1.1, respectively. Forty eight hours after transfection, cells were treated with puromycin at a concentration of 3 μg/ml for 2 days. Then, a single cell was seeded into 96-well plates and cultured for 1 month, and knockout of p53 protein was evaluated by western blot, and DNA sequencing was used for confirmation. For DNA sequencing, genomic DNA was extracted from cells, and a 383-bp polymerase chain reaction (PCR) amplicon flanking the CRSPR-Cas9-targeted sites (GCAGTCACAGCACATGACGG) was generated using the primers 5′-TCACTTACCTCTCAGAGAC-3′ (forward) and 5′-ACAGGGCAGGTCTTGGCCGTT-3′ (reverse). The PCR product was purified and ligated into the pMD18-T vector. The recombinant plasmids were introduced into competent DH5α cells. Plasmid DNA was extracted and sequenced across the insert using one of the PCR primers in the Life Technologies Corporation (Shanghai, China). The sequence of p53 KO CNE2 (KO-41) cells after knockout by CRISPR-Cas9 gene editing system was 5′-GGAGGCTACACGACACTGACGAACATC-3′. The sequence of p53 KO CNE2 (KO-49) cells after knockout by CRISPR-Cas9 gene editing system was 5′-GGAGGCAGGGGTCCGGAGACTAAGTACA-3′ and 5′- GGAGGCACTGACGAAC-3′. The sequence of p53 KO C666-1 (KO-9) cells after knockout by CRISPR-Cas9 gene editing system was 5′-GGAGTACACGACACTGACGAACATCTACCG-3′. The sequence of p53 KO C666-1 (KO-29) cells after knockout by CRISPR-Cas9 gene editing system was 5′-GGAGTACACGACACTGACGAACATCTACCG-3′.

### Western Blot

Proteins were exacted from cells using RIPA buffer, and subjected to SDS-PAGE separation, followed by blotting onto a PVDF membrane (Millipore, USA). Blots were incubated with antibodies against p53 (DO-1) (#sc-126, Santa Cruz, USA), phospho-AKT (Thr308; #4056, CST, USA), or AKT (#4691, CST, USA) overnight at 4°C, followed by incubation with HRP-conjugated secondary antibody for 1 h at room temperature. The signal was visualized with an enhanced chemiluminescence detection reagent (Millipore, USA).

### Detection of Heterozygous p53-R280T Mutation in NPC Cell Lines

Sanger sequencing was performed to detect the heterozygous R280T mutation of p53 gene using genomic DNA extracted from NPC CNE2, 5-8F, 6-10B, and C666-1 cell lines. Mutation was confirmed by at least two independent PCR amplifications and a DNA sequencing reaction on both strands. Oligonucleotide primers were designed to amplify exon 8 of p53 gene. The primers used were: 5′-GCTGGGGAGAGGAGCTGGTG-3′ (forward) and 5′-GGTTCATGCCGCCCATGCAG-3′ (reverse). The products were examined by sequencing in the Sangon Biotech, Shanghai, China.

#### 5-Ethynyl-2′-Deoxyuridine (EdU) Incorporation Assay

EdU incorporation assay was performed to detect cell proliferation as described previously by us (20). The assay was performed three times in triplicate.

### Cell Counting Kit-8 (CCK-8) Assay

Cell proliferation was measured using a CCK-8 kit as described previously by us (20). The assay was performed three times in triplicate.

### Flow Cytometry Analysis of Cell Cycle and Apoptosis

Flow cytometry analysis of cell cycle and apoptosis was performed as described previously by us (21). All assays were performed three times in triplicate.

### Anchorage Dependent and Independent Colony Formation Assay

Plate colony formation assay and soft agar colony formation assay were performed to detect the anchorage dependent or independent growth of cells as described previously by us (22). All assays were performed three times in triplicate.

### Tumor Formation Assay in Nude Mice

Nude male mice that were 4 weeks old were obtained from the Laboratory Animal Center of Central South University (Changsha, China) and were maintained under specific pathogen-free conditions. 5 × 10^6^ cells resuspended in 200 μl of serum-free medium were subcutaneously injected into the flanks of mice (*n* = 3 mice each). The mice were monitored daily for palpable tumor formation, and tumor volume (in mm^3^) was measured by a vernier caliper every 3 days and calculated by using the modified ellipse formula (volume = length × width^2^/2). At the end of the experiments, the mice were killed by cervical dislocation, and tumors were excised, and weighted.

### mRNA Sequencing

Total RNA was extracted from NPC cells with Trizol reagent (Invitrogen, USA). Two microgram RNA per sample was used as input material for the RNA sample preparations. Sequencing libraries were generated using NEBNext® Ultra™ RNA Library Prep Kit for Illumina® (#E7530L, NEB, USA), and index codes were added to attribute sequences to each sample. Briefly, mRNA was purified from total RNA using poly-T oligo-attached magnetic beads. First strand cDNA was synthesized using random hexamer primer and RNase H. Second strand cDNA synthesis was subsequently performed using buffer, dNTPs, DNA polymerase I and RNase H. The library fragments were purified with QiaQuick PCR kits and elution with EB buffer, then terminal repair, A-tailing and adapter added were implemented. The aimed products were retrieved and PCR was performed, then the library was completed. The libraries were sequenced on an Illumina platform and 150 bp paired-end reads were generated. Reads count for each gene in each sample was counted by HTSeq v0.6.0, and FPKM (Fragments Per Kilobase Millon Mapped Reads) was then calculated to estimate the expression level of genes in each sample. DESeq (v1.16) was used for differential gene expression analysis between two samples with biological replicates using a model based on the negative binomial distribution. The DEGs standard is (|log_2_ Fold change|≥2, and *q* < 0.05). The GO enrichment of differentially expressed genes (DEGs) was implemented by the hypergeometric test, in which *p*-value is calculated and adjusted as *q*-value, and data background is genes in the whole genome. GO terms with *q* < 0.05 were considered to be significantly enriched. The KEGG enrichment of DEGs was implemented by the hypergeometric test. KEGG terms with *p* < 0.05 were considered to be significantly enriched.

### qRT-PCR

Total RNA was extracted from NPC cells with Trizol reagent (Invitrogen, USA). One microgram of total RNA was reversely transcribed for cDNA using a RT kit according to the manufacturer's protocol and Oligo dT primer (Vazyme Biotech, China) according to the manufacturer's instruction. The RT products were amplified by real-time PCR using SYBR qPCR Master Mix kit (Vazyme Biotech, China) according to the manufacturer's instruction. The products were quantitated using 2^−DDCt^ method against GAPDH for normalization. The primer sequences were synthesized by the Sangon Biotech (Shanghai, China) and listed in Supplementary Table S1.

### Statistical Analysis

All the quantified data represented an average of three times. Data are represented as mean ± SD. One-way analysis of variance or two-tailed Student's *t*-test was used for comparisons between groups. Differences were considered statistically significant when *P* < 0.05.

## Results

### Heterozygous p53-R280T Mutation Occurs in NPC Cell Lines

**Figure 1:**
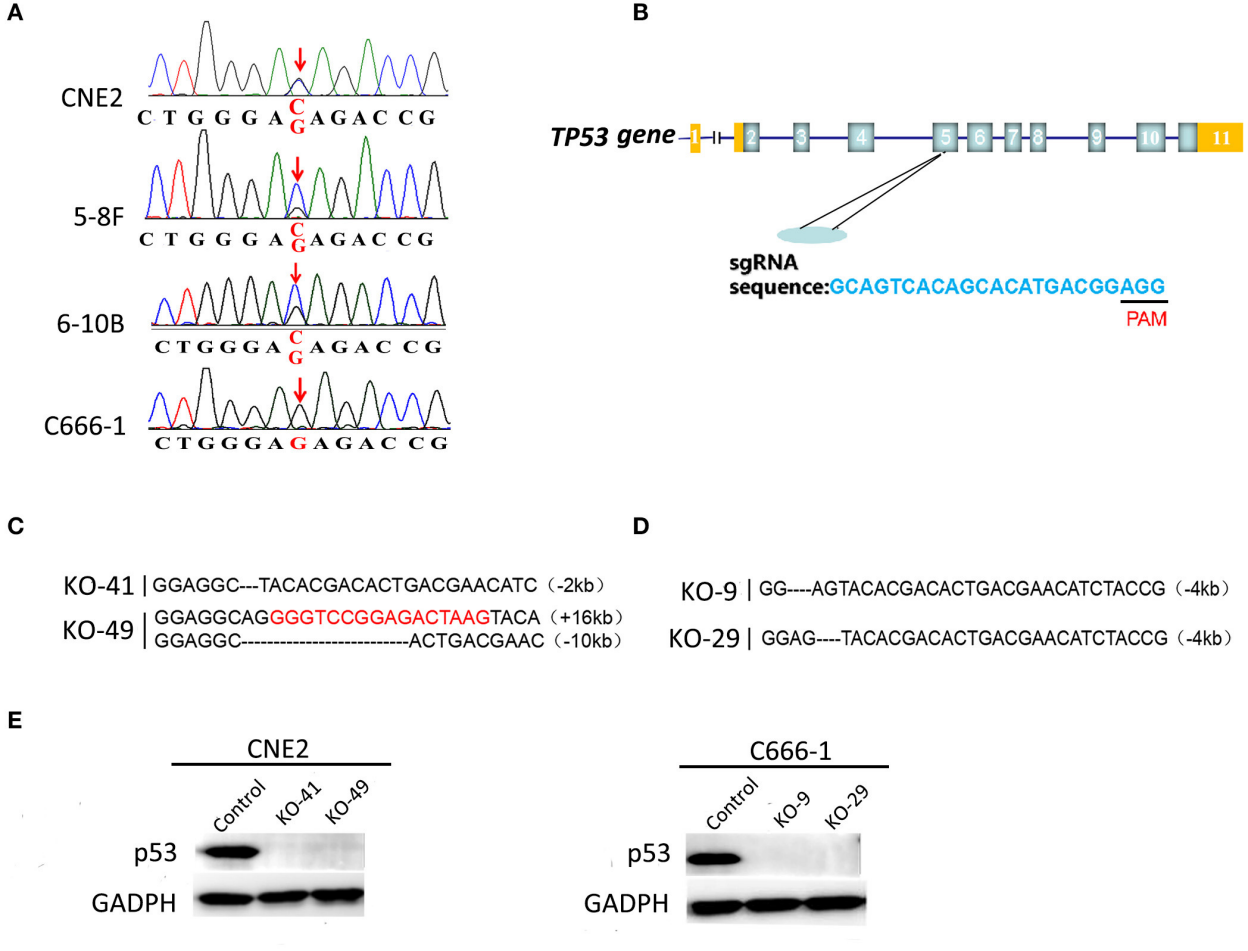
Detection of heterozygous p53-R280T mutation and generation of p53 knockout NPC cell lines using CRISPR/Cas9 gene editing system. **(A)** DNA sequencing showing heterozygous R280T mutation in CNE2, 5–8F, 6–10B but not C666-1 cells. **(B)** The gene structure of p53 in human genome (top) and single guide RNA (sgRNA) target sequence in p53 loci (bottom) are shown. PAM: protospacer adjacent motif (black bar). **(C)** Alignment analysis of the nucleic acid sequences of p53 gene in the knockout (KO) CNE2 cells. The black characters “-” indicate the deleted bases of p53 gene. The inserted bases are highlighted in red. **(D)** Alignment analysis of the nucleic acid sequences of p53 gene in the KO C666-1 cells. The black characters “-” indicate the deleted bases of p53 gene. **(E)** Western blot analysis showing p53 expression levels in the p53 KO CNE2 (KO-41, KO49) and p53 KO C666-1 cells (KO-9 and KO-29) and their control cells. KO, p53 knockout.

Genomic DNA obtained from CNE2, 5-8F, 6-10B, and C666-1 cells was amplified and detected for mutations at codon 280 of p53 gene by Sanger sequencing. Alignment analysis of DNA sequences was performed using the NCBI BLAST. A heterozygous G changed to C point mutation at codon 280, position 2 (AGA coding for arginine changed to ACA coding for threonine) was detected in the CNE2, 5-8F, 6–10B cell lines (Figure 1A), which indicated that one allele was mutated, the other allele was retained as normal at codon 280. However, the amplified DNA sequences of p53 at codon 280 from C666-1 cells were exactly the same as the human wild-type (wt) p53 sequences, compared with the database (Figure 1A). The results confirmed that heterozygous p53-R280T mutation is present in CNE2, 5-8F and 6-10B cells, but not in C666-1 cells.

### Generation of p53 Knockout NPC Cell Lines by CRISPR/Cas9 Gene Editing System

To study the roles of heterozygous p53-R280T mutation in NPC cells, we established p53 knockout (KO) CNE2 and C666-1 cell lines, in which p53 was knocked out at the chromosomal level by using CRISPR/Cas9 gene editing system. Single-guide RNA (sgRNA) was designed to delete exon 5 of the p53 gene (Figure 1B). Guide RNA (gRNA) vector and control vector were transfected into CNE2 and C666-1 cells, respectively. Sanger sequencing was used to identify the cell lines in which both alleles of p53 were deleted. The results showed that p53 was knocked out in the CNE2 cells (KO-41 and KO-49) (Figure 1C), and C666-1 cells (KO-9 and KO-29) (Figure 1D). Western blot analysis showed that there was no detectable p53 protein in the p53 KO CNE2 and C666-1 cells (Figure 1E). The results demonstrated that CNE2 and C666-1 cell lines with p53 KO are established.

### Heterozygous p53-R280T Mutation Promotes NPC Cell Proliferation and Survival

**Figure 2:**
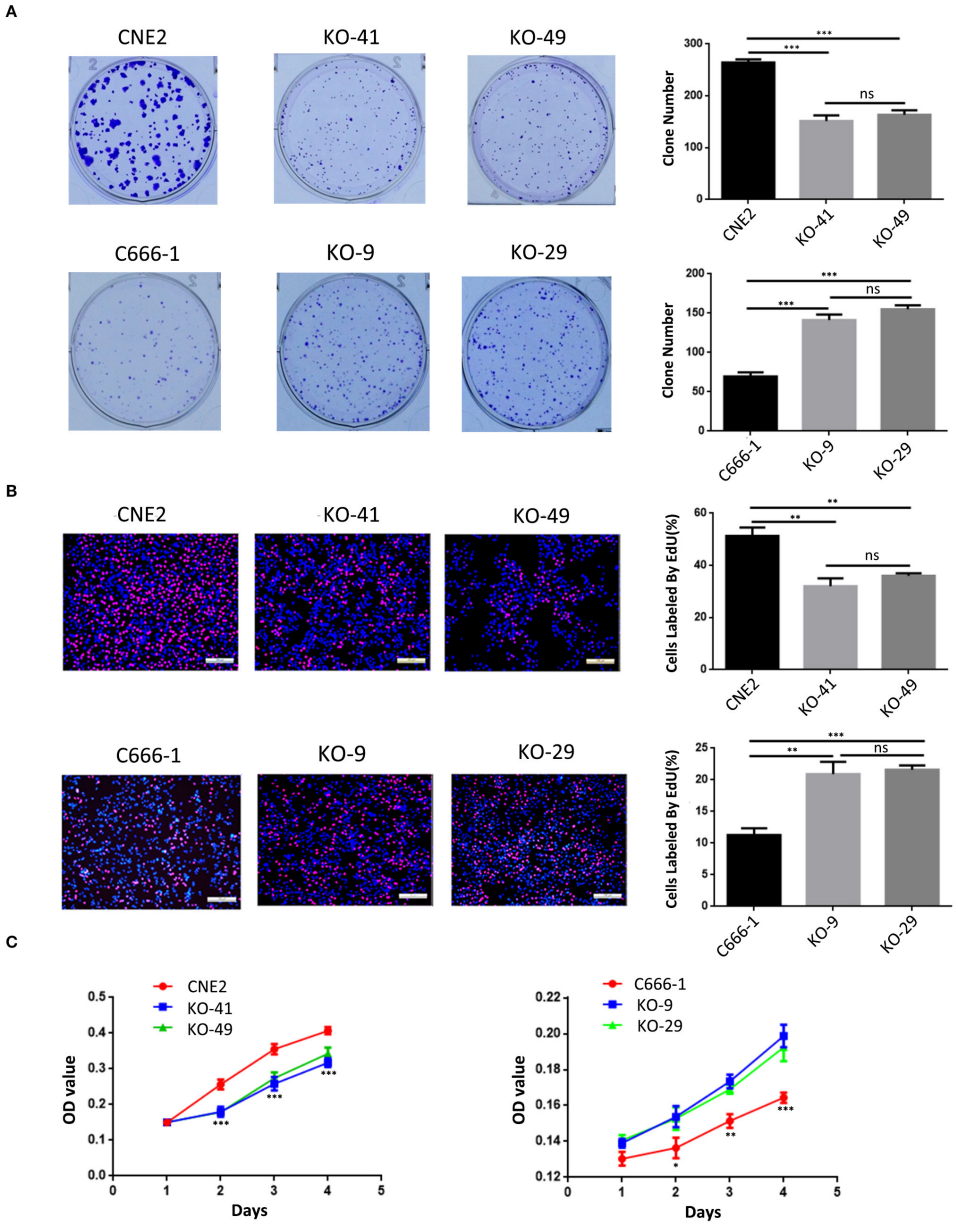
The effect of heterozygous p53-R280T and wt p53 knockout on NPC cell proliferation. **(A,B)** Representative results (left) and statistical analyses (right) of cell proliferation detected by plate clone formation assay and EdU incorporation assay in the p53 KO CNE2 and C666-1 cells and their control cells. **(C)** CCK-8 assay showing cell proliferation in the p53 KO CNE2 and C666-1 cells and their control cells. *P < 0.05; **P < 0.01; ***P < 0.001; ns, no significance. KO, p53 knockout.

**Figure 3:**
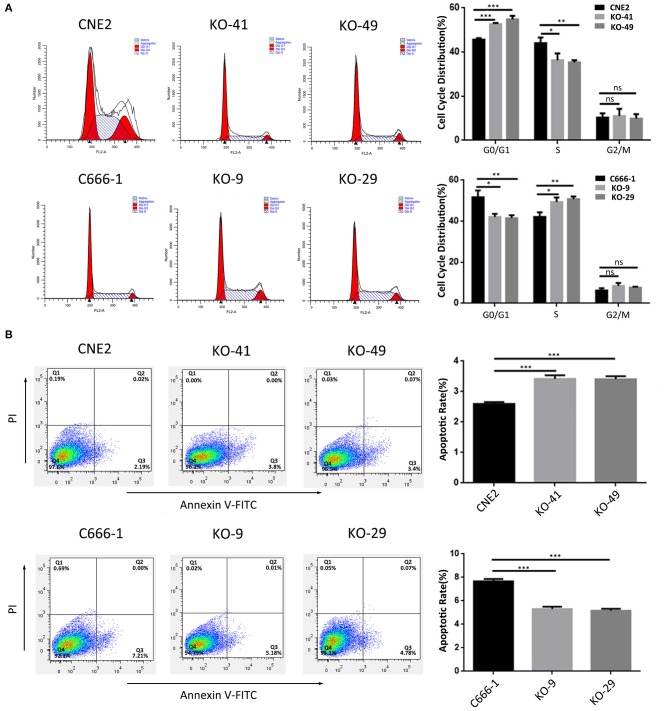
The effect of heterozygous p53-R280T and wt p53 knockout on cell cycle and apoptosis of NPC cells. **(A)** Representative results (left) and statistical analyses (right) of cell cycle distribution analyzed by flow cytometry in the p53 KO CNE2 and C666-1 cells and their control cells. **(B)** Representative results (left) and statistical analyses (right) of cell apoptosis analyzed by flow cytometry in the p53 KO CNE2 and C666-1 cells and their control cells. *P < 0.05; **P < 0.01; ***P < 0.001; ns, no significance. KO, p53 knockout.

We evaluated the effect of p53 KO on NPC cell proliferation by plate colony formation assay, EdU incorporation assay and CCK-8 assay. The results showed that p53 KO significantly suppressed cell proliferation in the CNE2 cells with heterozygous p53-R280T mutation, whereas significantly promoted cell proliferation in C666-1 cells with wt p53 (Figures 2A–C). Flow cytometric analysis of cell cycle distribution showed that p53 KO blocked G1/S phase progression in the CNE2 cells, whereas accelerated G1/S phase progression in the C666-1 cells (Figure 3A). Next, we analyzed the effect of p53 KO on the apoptosis of CNE2 and C666-1 cells by using flow cytometry. The results showed that p53 KO significantly increased cell apoptosis in the CNE2 cells, whereas significantly decreased cell apoptosis in the C666-1 cells (Figure 3B). Together, the results demonstrated that heterozygous p53-R280T mutation promotes NPC cell proliferation and survival.

### Heterozygous p53-R280T Mutation Promotes Anchorage-Independent Growth and *in vivo* Tumorigenicity of NPC Cells

**Figure 4:**
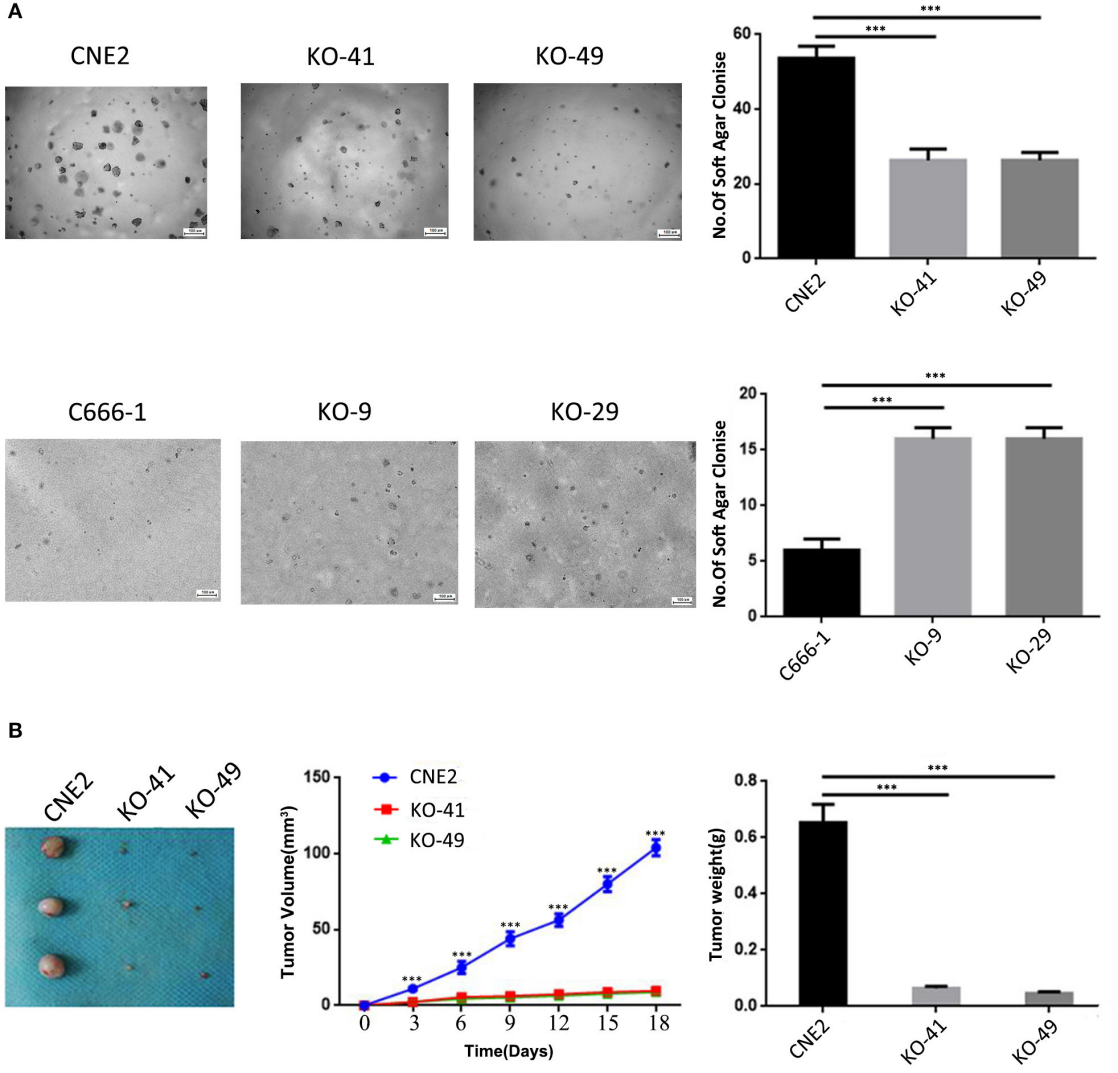
The effects of heterozygous p53-R280T and wt-p53 knockout on anchorage-independent and xenograft growth of NPC cells. **(A)** Representative results (left) and statistical analyses (right) of soft agar colony formation ability in p53 KO CNE2 and C666-1 cells and their control cells. **(B)** The photography of xenograft tumors 18 days after subcutaneous implantation of p53 KO CNE2 cells and control cells (left), and growth curve and weight of xenograft tumors generated by p53 KO CNE2 cells and control cells (middle and right). *n* = 3 mice per group. ***P < 0.001. KO, p53 knockout.

Soft agar colony formation assay and subcutaneous tumor formation experiment in nude mice were performed to determine the effects of heterozygous p53-R280T mutation on the anchorage-independent growth and *in vivo* tumorigenicity of NPC cells respectively. The results showed that p53 KO significantly decreased the formation ability of soft agar colony in the CNE2 cells, whereas significantly increased the formation ability of soft agar colony in the C666-1 cells (Figure 4A). Subcutaneous tumor formation experiment showed that p53 KO significantly decreased the *in vivo* growth of CNE2 cells in nude mice (Figure 4B). The results demonstrated that heterozygous p53-R280T mutation promotes the anchorage-independent growth and *in vivo* tumorigenicity of NPC cells.

### Differentially Expressed Genes in the Heterozygous p53-R280T KO and Control CNE2 Cells

**Figure 5:**
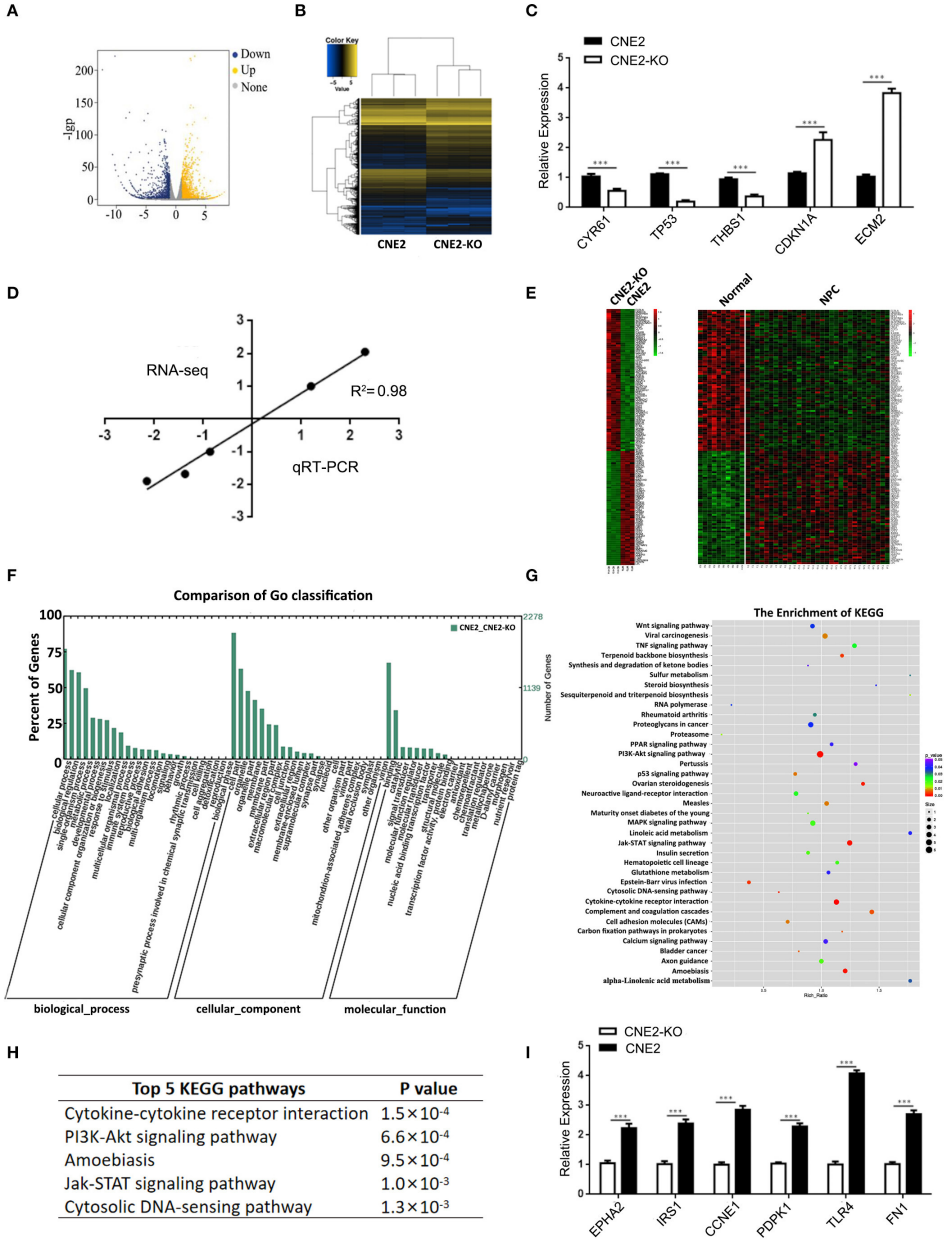
Differentially expressed genes in the heterozygous p53-R280T KO CNE2 and control CNE2 cells. **(A)** mRNA-sequencing showing differentially expressed genes that change more than 2-fold in the p53 KO CNE2 and control CNE2 cells. Blue dots represent down-regulated genes and yellow dots represent up-regulated genes. **(B)** Hierarchical clustering of differentially expressed genes in the p53 KO CNE2 and control CNE2 cells. **(C)** qRT-PCR detection of the five differentially expressed genes identified by mRNA sequencing in the p53 KO CNE2 and control CNE2 cells. **(D)** Correlation of Log_2_ fold change derived from mRNA sequencing with the Log_2_ fold change obtained from qRT-PCR. **(E)** Integrated analysis of differentially expressed genes in p53 KO CNE2 vs. control CNE2 cells and primary NPCs vs. normal nasopharyngeal mucosal tissues. **(F)** GO enrichment analysis of differentially expressed genes in the p53 KO CNE2 and control CNE2 cells according to biological process, cellular component and molecular function. **(G)** KEGG enrichment analysis of the differential expression genes in p53 KO CNE2 and control CNE2 cells. **(H)** Top 5 KEGG pathways of enriched differentially expressed genes in the p53 KO CNE2 and control CNE2 cells. **(I)** qRT-PCR detection of 6 differentially expressed genes mRNA levels (EPHA2, IRS1, CCNE1, PDPK1, TLR4, and FN1) that enriched in PI3K-Akt signaling pathway and downregulated in the p53 KO CNE2 cells. ***P < 0.001. KO, p53 knockout; Normal, normal nasopharyngeal mucosal tissue.

To explore the mechanism by which heterozygous p53-R280T mutation promotes the oncogenicity of NPC cells, we carried out mRNA sequencing in the p53 KO CNE2 (KO-41) and control CNE2 cells. As a result, a total of 2,612 differentially expressed genes (DEGs) (fold change≥2) were identified in the KO-41 and control CNE2 cells (Figures 5A,B). Of them, 1401 DEGs were upregulated and 1211 DEGs were downregulated in the KO-41 cells (Supplementary Table S2). To verify the mRNA sequencing results, qRT-PCR was conducted to detect the mRNA levels of 5 genes (CYR61, TP53, THBS1, CDKN1A, and ECM2) in the KO-41 and control CNE2 cells. The results showed that the relative expression patterns of the five genes were consistent with mRNA sequencing data, with a correlation coefficient of 0.98 between qRT-PCR and mRNA sequencing results (Figures 5C,D). The results demonstrated that the mRNA sequencing results are reliable.

To investigate whether the DEGs in the p53 KO and control CNE2 cells are abnormally expressed in NPC biopsies, we downloaded the gene expression profile of NPC tissues from the GEO database (GSE12452) (23), and compared the DEGs with the differential gene expression profile of NPC tissues. The result showed that sixty-one mRNAs, such as PCNA, TIGAR, MMP1, FN1, SPARC and POSTN, upregulated in the CNE2 cells were also upregulated in NPC tissues; seventy-six mRNAs, such as CDKN2B, BCL6, KLF4 and TP53INP2, downregulated in the CNE2 cells were also downregulated in NPC tissues (Figure 5E, Supplementary Table S3), which suggests that these DEGs regulated by heterozygous p53-R280T mutation maybe participate in the carcinogenesis of NPC.

### Gene Ontology and KEGG Pathways Enrichment Analysis of Differentially Expressed Genes

The 2612 DEGs identified in the present study were formulated into an XML-based input data set to query the GO database. The results showed that all DEGs were divided into three major groups: cellular component, molecular function and biological process, as well as 55 functional groups (Supplementary Table S4). In the cellular component, molecular function and biological process, 17, 14, and 24 functional groups were annotated respectively, many of which are involved in tumorigenesis, such as the regulation of cell growth, cell adhesion, antioxidant, and metabolic process (Figure 5F).

The 2612 DEGs were uploaded into the KEGG database for pathway enrichment analysis. The results showed that 37 pathways were found to be statistically enriched (Supplementary Table S5), including many pathways involved in tumor development and progression, such as cytokine-cytokine receptor interaction, PI3K-Akt signaling pathway, Jak-STAT signaling pathway and cytosolic DNA-sensing pathway (Figures 5G,H). Moreover, the expression of 66 genes in the PI3K signaling pathway was downregulated in the p53 KO CNE2 cells (Supplementary Table S6). qRT-PCR was performed to detect the mRNA levels of 6 differentially expressed genes (EPHA2, IRS1, CCNE1, PDPK1, TLR4, and FN1) that enriched in PI3K-Akt signaling pathway and downregulated in the p53 KO CNE2 cells. The results showed that the expression levels of these genes were reduced in the p53 KO CNE2 cells (Figure 5I). These results suggest that PI3K-Akt pathway signaling may be involved in heterozygous p53-R280T mutation-mediated NPC promotion.

### Activation of PI3K-Akt Signaling Pathway Is Involved in Heterozygous p53-R280T Mutation-Mediated NPC Promotion

**Figure 6:**
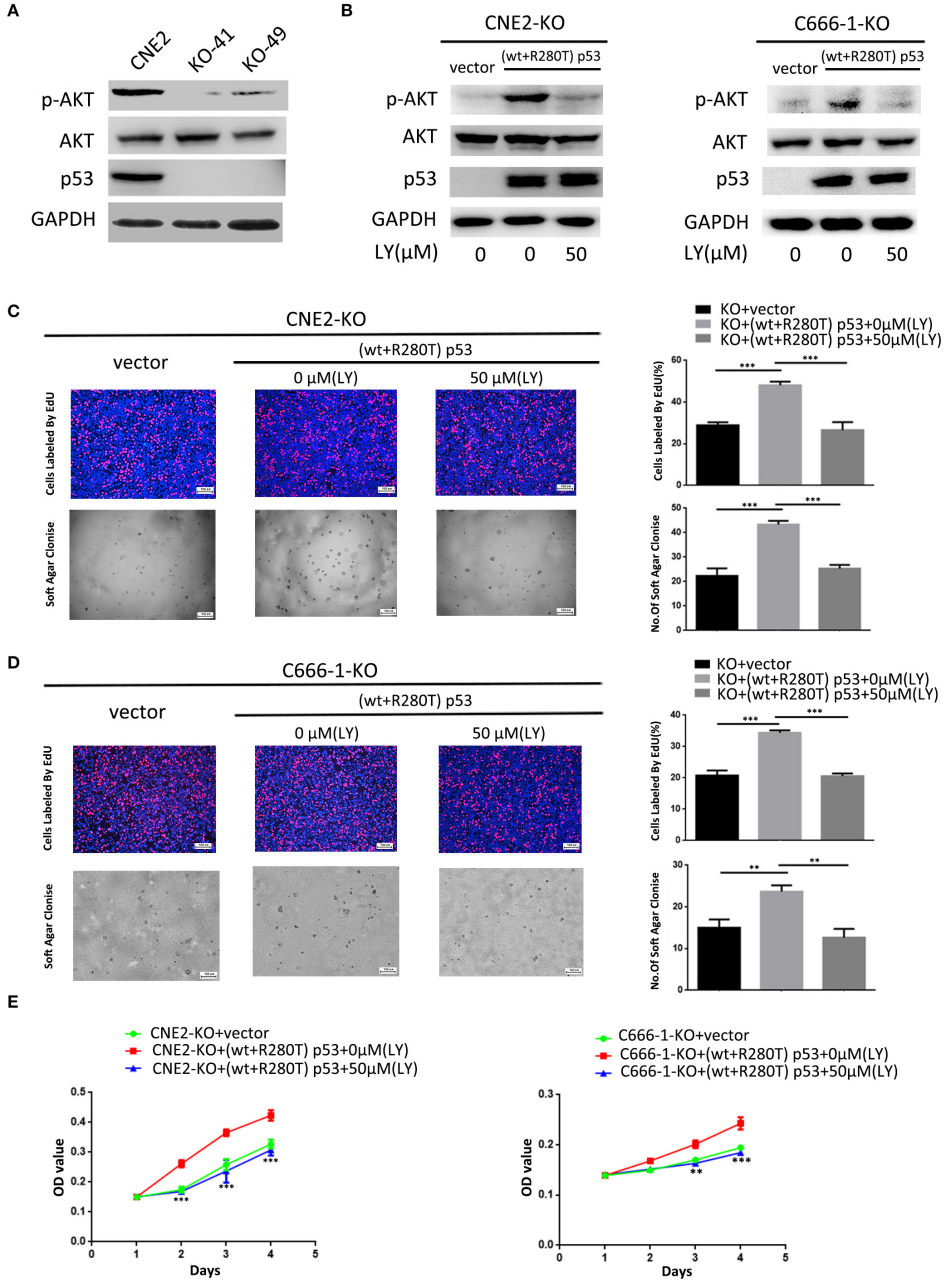
PI3K-Akt signaling pathway mediates heterozygous p53-R280T mutation-promoting NPC cell proliferation. **(A)** Western blot analysis showing the levels of p-AKT(S308) in the p53 KO CNE2 and control CNE2 cells. **(B)** Western blot analysis showing the levels of p-AKT(S308) in the p53 KO CNE2 and C666-1 cells transiently transfected with p53-R280T mutation and wt p53 plasmid at a 1:1 ratio, followed by treatment with 50 μM LY294002 for 12 h. **(C,D)** Representative results (left) and statistical analyses (right) of cell proliferation detected by EdU incorporation assay and soft agar colony formation assay in the p53 KO CNE2 and C666-1 cells transiently transfected with p53-R280T mutation and wt p53 plasmid at a 1:1 ratio, followed by treatment with 50 μM LY294002 for 12 h. **(E)** CCK-8 assay showing cell proliferation in the p53 KO CNE2 and C666-1 cells transiently transfected with p53-R280T mutation and wt p53 plasmid, followed by treatment with 50 μM LY294002 for 12 h. **P < 0.01, ***P < 0.001. Vector, transfected with an empty vector; KO, p53 knockout; LY, LY294002.

**Figure 7:**
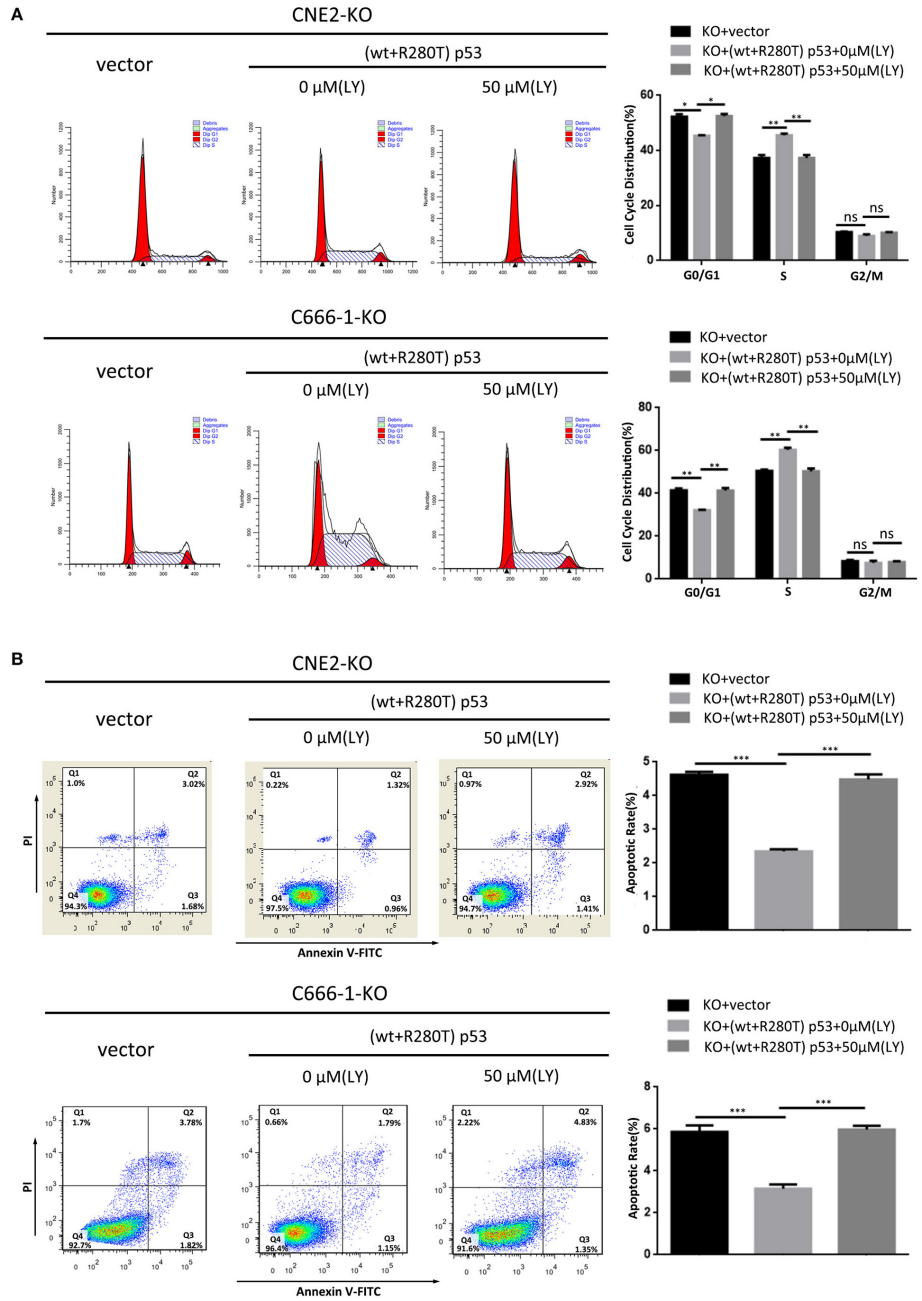
PI3K-Akt signaling pathway mediates the effect of heterozygous p53-R280T on cell cycle and apoptosis of NPC cells. **(A)** Representative results (left) and statistical analyses (right) of cell cycle distribution analyzed by flow cytometry in the p53 KO CNE2 and C666-1 cells transiently transfected with p53-R280T mutation and wt p53 plasmid at a 1:1 ratio, followed by treatment with 50μM LY294002 for 12h. **(B)** Representative results (left) and statistical analyses (right) of cell apoptosis analyzed by flow cytometry in the p53 KO CNE2 and C666-1 cells transiently transfected with p53-R280T mutation and wt p53 plasmid at a 1:1 ratio, followed by treatment with 50 μM LY294002 for 12h. *P < 0.05; **P < 0.01; ***P < 0.001; ns, no significance. Vector, transfected with an empty vector; KO, p53 knockout; LY, LY294002.

To investigate whether PI3K-Akt signaling pathway is involved in heterozygous p53-R280T mutation-mediated NPC promotion, we detected the levels of p-AKT in the KO-41, KO-49, and control CNE2 cells by western blot, and observed that p-AKT was significantly decreased in the KO-41 and KO-49 cells relative to control CNE2 cells (Figure 6A). Moreover, we transiently transfected p53 KO CNE2 and p53 KO C666-1 cells with wt p53 and p53-R280T mutation plasmid at 1:1 ratio (equal to transfection of heterozygous p53-R280T mutation), and then treated the cells with PI3K inhibitor LY294002. We observed that transfection of wt p53 and p53-R280T mutation plasmid at 1:1 ratio dramatically increased p-AKT levels in the p53 KO CNE2 and p53 KO C666-1 cells, which was abolished by LY294002 (Figure 6B). The results demonstrated that heterozygous p53-R280T mutation activated PI3K-Akt signaling pathway in NPC cells. Functionally, transfection of wt p53 and p53-R280T mutation plasmid at 1:1 ratio promoted *in vitro* cell proliferation of p53 KO CNE2 and p53 KO C666-1 cells, which was abolished by LY294002 (Figures 6C–E). Moreover, flow cytometric analysis showed that transfection of wt p53 and p53-R280T mutation plasmid at 1:1 accelerated G1/S phase progression and inhibited cell apoptosis in the p53 KO CNE2 and p53 KO C666-1 cells, which was abolished by LY294002 (Figures 7A,B). These results indicate that PI3K-Akt signaling pathway activation is involved in the tumor-promoting effects of heterozygous p53-R280T mutation in NPC cells.

## Discussion

In 1992, a heterozygous p53-R280T mutation was first detected in the NPC CNE2 and CNE1 cell lines (17). Consistent with this report, we also observed the same heterozygous mutation of p53 gene in the NPC CNE2, 5-8F, and 6-10B cell lines by Sanger sequencing. Previous study has shown that p53 gene is wild-type in the NPC C666-1 cell lines (15). We also did not find p53-R280T mutation in the C666-1 cells. The heterozygous p53-R280T mutation also exists in NPC tissues, with a mutation rate of about 10% (17). It is suggested that p53-R280T mutation may occur in primary tumors or may be acquired during the establishment or culture of cancer cell lines *in vitro* (17, 24). Nonetheless, the biological functions of heterozygous p53-R280T mutation in cancers remain unclear. To determine the roles of heterozygous p53-R280T mutation in NPC, we chose CNE2 with heterozygous R280T mutation and C666-1 with wt p53 gene to establish p53 knockout cell lines. We found that knockout of endogenous p53 gene with heterozygous p53-R280T mutation suppressed NPC proliferation and increased NPC cell apoptosis, and inhibited the anchorage-independent growth and *in vivo* tumorigenicity of NPC cells. In contrast, knockout of wt p53 had the opposite effects on NPC cells. Moreover, transfection of wt p53 and p53-R280T mutation plasmid at 1:1 ratio, which is equal to heterozygous p53-R280T mutation, promoted NPC cell proliferation and survival in the NPC cells with endogenous p53 knockout. Our results indicate that heterozygous p53-R280T mutation gains oncogenic activities and functions as an oncogene in NPC cells.

To explore the mechanism by which heterozygous p53-R280T mutation is involved in the tumor promotion of NPC cells, we carried out mRNA sequencing in the p53 KO and control CNE2 cells, and observed that 1401 DEGs were upregulated, and 1211 DEGs were downregulated in the p53 KO CNE2 with heterozygous p53-R280T mutation. To investigated whether these DEGs were abnormally expressed in NPC biopsies, we compared these DEGs with the differential gene expression profile from NPC biopsies (GSE12452) (23), and observed that 61 mRNAs upregulated in the CNE2 cells were also upregulated in the NPC biopsies, and 66 mRNAs downregulated in the CNE2 cells were also downregulated in the NPC biopsies, suggesting that these DEGs regulated by heterozygous p53-R280T mutation maybe participate in the carcinogenesis of NPC. Besides, KEGG pathway enrichment analysis showed that the DEGs were statistically enriched in pathways related to cancer such as PI3K-Akt signaling pathway, Jak-STAT signaling pathway, MAPK signaling pathway, TNF signaling pathway and Wnt signaling pathway, which may be associated with the NPC promotion of heterozygous p53-R280T mutation. We also observed that the mRNA level of p53 target gene CDKN1A (p21) increased in the p53 KO CNE2 cells with heterozygous p53-R280T mutation. Previous report also shows that p53 silencing resulted in upregulation of p21 in CNE2 cells (25), supporting that heterozygous p53-R280T mutation gains oncogenic property in NPC cells.

PI3K plays an important role in cancer development and progression (26–32). Once activated, PI3K converts membrane-bound phosphatidylinositol 4, 5-biphosphate (PIP2) into phosphatidylinositol 3,4,5-triphosphate (PIP3) (33). PIP3 then recruits phosphoinositide-dependent kinase 1 (PDK1) to phosphorylate Akt at threonine 308. Subsequently, mTOR complex 2 (mTORC2) phosphorylates Akt at serine 473(Ser473) for AKT activation (34, 35). Thereafter, activated Akt interacts with downstream target proteins to regulate multiple biological processes. In the present study, mRNA sequencing of heterozygous p53-R280T KO CNE2 and control cells showed that heterozygous p53-R280T mutation activated PI3K-Akt signaling pathway, and transfection of wt p53 and p53-R280T mutation plasmid at 1:1 ratio dramatically increased p-AKT levels in the NPC cells with endogenous p53 KO, which was abolished by LY294002. The results demonstrated that heterozygous p53-R280T mutation activates PI3K-Akt signaling pathway in NPC cells. Importantly, blocking of PI3K-Akt signaling pathway abolished heterozygous p53-R280T mutation-promoting NPC cell proliferation and survival, indicating that heterozygous p53-R280T mutation promotes the oncogenicity of NPC cells by activating PI3K-Akt signaling pathway. Moreover, the genes enriched in PI3K-Akt signaling pathway, such as EPHA2, IRS1, FN1, PDGFRB, THBS1, CCND1, CCNE1, TLR4, FGFR1 and FLT1, promote the development and progression of NPC (36–46). Therefore, heterozygous p53-R280T mutation-activated PI3K-Akt signaling pathway may be involved in NPC carcinogenesis through theses target genes.

In summary, our data suggest that heterozygous p53-R280T mutation functions as an oncogene in NPC, and promotes the oncogenicity of NPC cells by activating PI3K-Akt signaling pathway. P53 knockout NPC cell lines and heterozygous p53-R280T mutation-associated DEGs provide a valuable tool to investigate the role and molecular mechanism of heterozygous p53-R280T mutation in NPC.

## Data Availability Statement

The datasets of mRNA-sequencing for this study can be found in the GEO: http://www.ncbi.nlm.nih.gov/geo/query/acc.cgi?acc=GSE130398.

## Ethics Statement

All animal experimental procedures were performed in accordance with the Guide for the Care and Use of Laboratory Animals of Xiangya Hospital, Central South University, with the approval of the Institutional Animal Ethics Committee.

## Author Contributions

Z-QQ and Z-QX designed experiments. Z-QQ, Q-GL, and HY implemented experiments. Z-QQ, Z-QX, Q-GL, WH, S-SL, Y-YT, and Z-XR analyzed experimental results. ZQ-Q and Z-QX wrote the manuscript.

### Conflict of Interest

The authors declare that the research was conducted in the absence of any commercial or financial relationships that could be construed as a potential conflict of interest. The reviewer GL declared a shared affiliation, though no other collaboration, with the authors to the handling Editor.
