# Supplementary material for: Heterozygous p53-R280T Mutation Enhances the Oncogenicity of NPC Cells Through Activating PI3K-Akt Signaling Pathway
Source: Front Oncol. 2020 Feb 5;10:104. doi: 10.3389/fonc.2020.00104 (PMC7025553; doi:10.3389/fonc.2020.00104)
Supplement: Supplementary file 1 [file Table_1.DOC]

| No. | Gene name | GenBank  Accession No. | Primer sequences |
| --- | --- | --- | --- |
| 1 | CYR61 | [NM_001554.5](https://www.ncbi.nlm.nih.gov/nuccore/NM_001554.5) | 5’-CTTGTGAAAGAAACCCGGATTT-3’ (forward) |
|  |  |  | 5’-ACTCAAACATCCAGCGTAAGTA-3’ (reverse) |
| 2 | TP53 | [NM_001126114.2](https://www.ncbi.nlm.nih.gov/entrez/viewer.fcgi?db=nucleotide&id=371502117) | 5’-TTCCTGAAAACAACGTTCTGTC-3’ (forward) |
|  |  |  | 5’-AACCATTGTTCAATATCGTCCG-3’ (reverse) |
| 3 | THBS1 | [NM_003246.4](https://www.ncbi.nlm.nih.gov/entrez/viewer.fcgi?db=nucleotide&id=1653961217) | 5’-CATCTTGTTCTGTGACATGTGG-3’(forward) |
|  |  |  | 5’-TTCACAGGGTTTCCCGTTC-3’ (reverse) |
| 4 | CDKN1A | NM_001220778.1 | 5’- GATGGAACTTCGACTTTGTCAC-3’ (forward) |
|  |  |  | 5’- GTCCACATGGTCTTCCTCTG-3’ (reverse) |
| 5 | ECM2 | NM_001393.3 | 5’-CCGAATGCCCTCTCGATCC-3’ (forward) |
|  |  |  | 5’-TGGGTAAGCATGGCGTTGATG-3’ (reverse) |
| 6 | EPHA2 | NM_004431.5 | 5’- TGAATGACATGCCGATCTACAT-3’ (forward) |
|  |  |  | 5’- CTTGAGCTCAATGAAGATACGC-3’ (reverse) |
| 7 | IRS1 | NM_005544.2 | 5’- TCATCTCCTCGGATGAGTATGG-3’ (forward) |
|  |  |  | 5’- ACCCATGCAGATATAGTTGCTT-3’ (reverse) |
| 8 | CCNE1 | NM_001238.4 | 5’- TTGTGTCCTGGCTGAATGTATA-3’ (forward) |
|  |  |  | 5’- AAGGAAATTCAAGGCAGTCAAC-3’ (reverse) |
| 9 | PDPK1 | NM_002613.5 | 5’- AAGGGTTTATTTGCAAGACGAC-3’ (forward) |
|  |  |  | 5’- GAACTTTGTTGACAGGATCCAC-3’ (reverse) |
| 10 | FN1 | NM_212482.3 | 5’- AATAGATGCAACGATCAGGACA-3’ (forward) |
|  |  |  | 5’- GCAGGTTTCCTCGATTATCCTT-3’ (reverse) |
| 11 | TLR4 | NM_138554.5 | 5’- GACTGGGTAAGGAATGAGCTAG-3’ (forward) |
|  |  |  | 5’- ACCTTTCGGCTTTTATGGAAAC-3’ (reverse) |
| 12 | GAPDH | [NM_001256799.3](https://www.ncbi.nlm.nih.gov/entrez/viewer.fcgi?db=nucleotide&id=1676318038) | 5’-CTTTGTCAAGCTCATTTCCTGGTA-3’ (forward) |
|  |  |  | 5’-GGCCATGAGGTCCACCA-3’ (reverse) |

**Supplementary Table S1. The primers used for detecting the mRNAs by qRT-PCR**
